# Wearable Electrochemical Glove-Based Analytical Device (eGAD) for the Detection of Methamphetamine Employing Silver Nanoparticles

**DOI:** 10.3390/bios13100934

**Published:** 2023-10-18

**Authors:** Nigar Anzar, Shariq Suleman, Yashda Singh, Suhel Parvez, Manika Khanuja, Roberto Pilloton, Jagriti Narang

**Affiliations:** 1 Department of Biotechnology, School of Chemical and Life Science, Jamia Hamdard University, New Delhi 110062, India; nigarsheikh111@gmail.com (N.A.); shariqsuleman07@gmail.com (S.S.); yashusingh2398@gmail.com (Y.S.); 2 Department of Toxicology, School of Chemical and Life Science, Jamia Hamdard University, New Delhi 110062, India; sparvez@jamiahamdard.ac.in; 3 Centre for Nanoscience and Nanotechnology, Jamia Millia Islamia, New Delhi 110025, India; manikakhanuja@gmail.com; 4 Institute of Crystallography, National Research Council (CNR-IC), 00015 Rome, Italy

**Keywords:** gloves, electrochemical, methamphetamine, wearable

## Abstract

Illicit drug misuse has become a widespread issue that requires continuous drug monitoring and diagnosis. Wearable electrochemical drug detection devices possess the potential to function as potent screening instruments in the possession of law enforcement personnel, aiding in the fight against drug trafficking and facilitating forensic investigations conducted on site. These wearable sensors are promising alternatives to traditional detection methods. In this study, we present a novel wearable electrochemical glove-based analytical device (eGAD) designed especially for detecting the club drug, methamphetamine. To develop this sensor, we immobilized meth aptamer onto silver nanoparticle (AgNPs)-modified electrodes that were printed onto latex gloves. The characteristics of AgNPs, including their shape, size and purity were analysed using FTIR, SEM and UV vis spectrometry, confirming the successful synthesis. The developed sensor shows a 0.1 µg/mL limit of detection and 0.3 µg/mL limit of quantification with a linear concentration range of about 0.01–5 µg/mL and recovery percentages of approximately 102 and 103%, respectively. To demonstrate its applicability, we tested the developed wearable sensor by spiking various alcoholic and non-alcoholic drink samples. We found that the sensor remains effective for 60 days, making it a practical option with a reasonable shelf-life. The developed sensor offers several advantages, including its affordability, ease of handling and high sensitivity and selectivity. Its portable nature makes it an ideal tool for rapid detection of METH in beverages too.

## 1. Introduction

Illicit drug use imposes both economic and social burdens on society, while also detrimentally impacting the health of individuals who use drugs [1]. Illicit drugs refer to substances that are illegal to produce, possess or distribute. The prevalence of illicit drug abuse has experienced a substantial rise in recent years, presenting a growing and significant challenge. This issue incurs an enormous cost exceeding USD 400 billion each year, which includes expenses related to reduced productivity, increased healthcare demands and legal proceedings [2,3]. In recent years, the misuse of prescription pain medications has reached epidemic proportions, posing a significant public health challenge. This includes the illicit diversion of commonly prescribed medications like methamphetamine, ketamine, oxycodone, buprenorphine, morphine, fentanyl, etc. [4,5]. Recent reports indicate that seizures caused by methamphetamine have been doubled over the last five years. The synthetic stimulant methamphetamine (meth) has an impact on the central nervous system. It is being misused more often, and the results are severe social and health issues. When in powder form it is referred as “crank” and when it is in pure crystal, it is called “ice” [6]. To address the prevention of its widespread transportation and abuse, various detection techniques have been developed, including gas chromatography (GC) [7], mass spectrometry (MS), liquid chromatography (LC) [8,9], capillary electrophoresis [10], immunoassays [11,12] and molecularly imprinted polymer solid-phase extraction [13,14,15,16]. These techniques aim to enhance the identification and control of meth distribution, effectively tackling its growth [17]. Nevertheless, these analytical methods are associated with drawbacks such as high costs, time-consuming processes and the need for skilled personnel [18,19,20]. Hence, there is an urgent need to develop a new approach that enables the rapid and sensitive detection of meth. Such advancements in this field are crucial to address the existing challenges and enhance efficiency in meth detection.

The field of digital health is witnessing a rapid emergence of wearable electrochemical sensors that enable the non-invasive monitoring of chemical markers; these sensors hold great promise in revolutionizing healthcare by providing real-time and convenient monitoring of various biomarkers for the improved diagnosis and management of health conditions [21,22]. The majority of wearable sensors have been developed with a focus on health, wellness and fitness, as well as security and forensic uses for these gadgets and the tracking of illicit drug usage [23,24]. Hand-worn gloves are a type of disposable wearable materials. Electrochemical sensor integration on gloves can open up novel possibilities in the fields of healthcare, environmental monitoring, food quality management and danger detection. Glove-based wearable chemical sensors provide exciting possibilities in a number of fields, such as quick, point-of-need chemical detection, and are primarily appropriate for defence, forensic and environmental applications [25,26,27].

Herein, we reported a wearable glove-based electrochemical analytical device (eGAD) for detecting methamphetamine. We worked by designing for the first time the combination of the flexible glove substrate and the exceptional electrical properties of silver nanoparticles. A meth-specific binding aptamer was also employed. Aptamers are nucleic acid sequences, either ribonucleic acid (RNA) or single-stranded deoxyribonucleic acid (ssDNA), that have capability to bind selectively to a wide number of targets. Their specific binding is determined by their unique three-dimensional structure. Aptamers can target various substances, including organic and inorganic molecules, peptides, proteins, etc. [28,29].

We cross-compared the working of the proposed sensor using a potentiostat via electrochemical techniques such as cyclic voltammetry (CV) and linear sweep voltammetry (LSV). The proposed sensor’s applicability was developed by spiking alcoholic and non- alcoholic drinks with meth. Further, the stability and repeatability of the sensor were also checked.

## 2. Experimental Method

### 2.1. Chemicals Used

All the chemicals used were obtained at their uppermost accessible quality from Sigma-Aldrich and were used as received, deprived of any additional purification. Carbon conductive ink and silver chloride paste were brought from Snab graphix Pvt Ltd., Bangalore, India, for fabricating electrodes. For synthesizing silver nanoparticles, silver nitrate, hydrochloric acid, sodium borohydride and sulphuric acid were bought from MTOR life science Pvt Ltd., Delhi, India. M=methylene blue and potassium chloride were purchased from Amplicon Biotech, Delhi, India.

Alcoholic (Whisky) and non-alcoholic drinks (Real juice) were taken for spike testing. All the drinks were purchased from local stores. Powder-free Rubber Latex Surgical Gloves were also purchased from local medical store, Delhi, India.

Methamphetamine was purchased from MTOR life science Pvt Ltd., Delhi, India. Aptamer sequence binding towards methamphetamine was purchased from Amplicon biotech, India.

Aptamer Sequence binding towards methamphetamine: (5′-ACG GTT GCA AGT GGG ACT CTG GTA GGC TGG GTT AAT TTG G-3′) [30].

### 2.2. Instrumentation and Measurements

The electrochemical profiles of the developed eGAD were recorded using the Metrohm Dropsens (stat-I 400s) instrument purchased from Metrohm Autolab B.V., Utrecht, The Netherland. Disposable glove-based electrodes were constructed by printing carbon conductive ink, while silver paste was used for the pseudo reference electrode. Electrochemical tests were conducted in a 10 mM KCl solution containing methylene blue at an appropriate pH. To examine the surface morphology of the material, Field Emission Scanning Electron Microscopy (FESEM) technology was employed, specifically the Quanta 3D FEG (FEI) model. The crystallinity of the synthesized nanoparticles was studied using X-ray diffraction (XRD) with the Rigaku Smart Cu Kα X-ray (1.540 Å) instrument. UV-Vis absorbance measurements were performed using the Agilent technologies, Cary100 series, and a UV-Vis spectrometer was utilized to determine the absorbance of the nanoparticles.

### 2.3. Silver Nanoparticles Synthesis

AgNPs were synthesized by employing a chemical method involving the reduction of silver ions into nanoscale silver particles using a reducing agent such as NaBH4. In this process, a freshly prepared 1 mM AgNO_3_ solution (1.6 mg in 10 mL distilled water) was slowly added drop by drop (at a rate of 1 drop per second) to a 2 mM ice-cold NaBH4 solution (2.26 mg in 30 mL distilled water), while continuously stirring the mixture. As the silver ions underwent reduction, the solution gradually changed its colour to a bright yellow, indicating the presence of AgNPs. Further, the solution was characterized with using UV-vis spectrometry, FESEM and FTIR [31].

### 2.4. Fabrication of eGAD and Modification of Electrodes with Nanoparticles and Aptamer

In order to facilitate hand printing, a screen with laser-cut electrode patterned solid skin was prepared. This screen was bonded to a fixed-size frame, forming a three-electrode setup. Carbon conductive ink was embraced on the latex gloves, forming electrodes by using a squeezer. The stencil on the silk screen defined the dimensions of the electrodes. The resulting printed electrodes comprise up of three components, i.e., the counter electrode (CE), working electrode (WE) and reference electrode (RE) drop-casted with Ag/AgCl. Ink-painted gloves were left to dry overnight, leading to the development of a glove biosensor based on a three-electrode system. This approach proposes many advantages with affordability, simplicity, reusability and a smaller sample volume. Additionally, the use of conductive carbon ink provides favourable characteristics such as cost-effectiveness, the ease of preparation and rapid fabrication.

**Figure 1:**
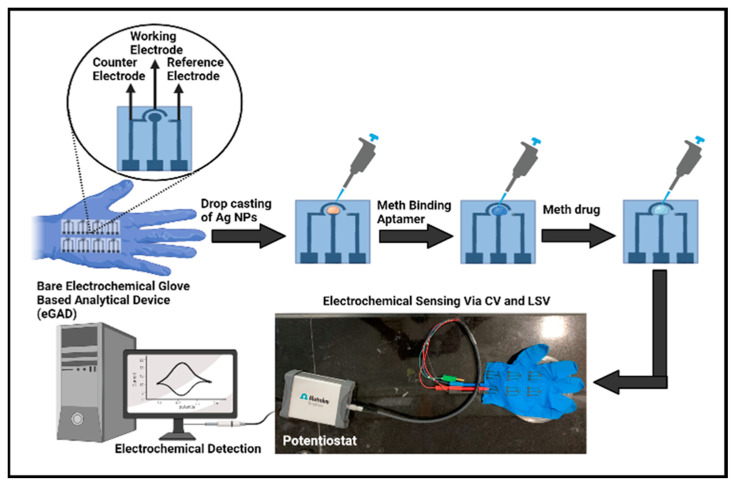
Graphical representation of electrochemical glove-based analytical device (eGAD) construction.

Figure 1 illustrates the various fabrication steps involved in the construction of an electrochemical glove-based analytical device (eGAD). In the first step, a fixed optimized amount 20 µL of AgNPs was drop-deposited onto the working region of an electrode. Further, the electrodes were dried overnight, ensuring proper nanoparticle adhesion. Once the silver nanoparticles were deposited, the working region was immobilized with a meth-specific aptamer of 20 µL volume.

To prepare the aptamer stock solutions, the dried aptamer pellet was reconstituted in a modified phosphate-buffered saline (PBS) at a pH of 7.5. The aptamer stock solution was first prepared at a concentration of 221 μL. It was then diluted to a working concentration of 1 μM in PBS. Subsequently, 20 µL of aptamer was drop-deposited onto the circular working area, specifically targeting the silver nanoparticles, and allowed to dry for a period of 4–5 h. These modified electrodes, featuring the combination of nanoparticles and aptamers, were designed for the recognition and detection of the target substance, meth, in subsequent analyses.

### 2.5. Detection Approach and Signal Procurement

**Figure 2:**
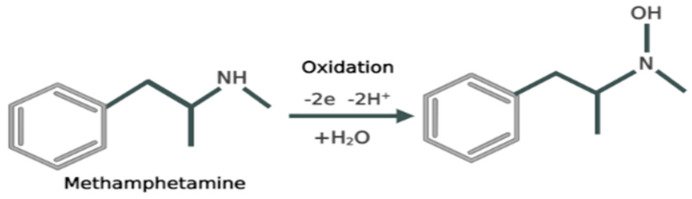
Reaction depicting the mechanism of electrochemical oxidation of meth.

Methamphetamine is a compound that exhibits electrochemical activity. The chemical makeup of meth indicates that the secondary amine in the aliphatic part of the molecule is likely group to undergo electro-oxidation. It engages the oxidation of primary and secondary amino groups as well as the oxidation of the aromatic nucleus [32]. The electrochemical oxidation process of methamphetamine involves several steps. Initially, the electrons are taken by the electrode from methamphetamine, leading to the formation of radical cations (METH•+). This radical cation undergoes rearrangement and possible bond cleavage, leading to the formation of intermediate species. Further oxidation reactions occur, involving the transfer of electrons and the potential participation of electrolyte species. These reactions result in the generation of final oxidation products, such as aldehydes, ketones, carboxylic acids or other oxidized derivatives. The specific details of the electrochemical oxidation process may vary depending on factors like the electrode material, electrolyte composition, pH and applied potential. Understanding this process is crucial for the electrochemical detection and analysis of meth [33,34] (Figure 2).

By introducing both the meth-specific aptamer and meth to eGAD, the electrochemical process is enhanced, leading to the amplification of the current response. As the concentration of meth increases, the current on the eGAD-sensing surface also increases. This increased current observed in the working region of the developed eGAD, combined with silver particles possessing a high surface area and rapid electron transfer kinetics, serves as a fundamental driving force. When a voltage is applied to the surface of the sensor, the modified eGAD containing the nanoparticles of silver and aptamer, meth undergoes oxidation.

### 2.6. Electrochemical Response Measurement of AgNPs/Apt/Meth on Gloves-Based Electrodes and Optimization of the Developed eGAD

The working electrode must have an aptamer attached to it with an addition of nanoparticles for developing a functional biosensor to detect meth. The CV and LSV values of bare electrodes without any deposition were recorded first. Then, after leaving the glove-based aptasensor to dry overnight, AgNPs were added, and both voltammetry tests were conducted. The glove-based aptasensor that contained dried AgNPs was used in the next stage, and CV/LSV values were recorded after the aptamer was attached to it. Meth was placed onto electrodes that contained both Ag NPs and an aptamer during the final stage of sensor development, and thereafter, the CV/LSV was carried out and recorded using methylene blue prepared in potassium chloride.

For the determination of the linear concentration range, we used diverse concentrations of meth. A concentration of 0.01–5 µg/mL was prepared from a stock solution (1.0 mg/mL) of meth, which was obtained from the company. A fixed concentration of meth to be determined was dropped on the circular region on Apt/AgNPs eGAD. The same pattern was followed with all the other concentrations as with a range of 0.01–5 µg/mL. Further, to optimize the performance of the sensor, the temperature (10–50 °C) and time of incubation (5–30 s) of meth/apt/AgNPs eGAD were examined by observing the variations in the voltagrams obtained in the different detection settings.

### 2.7. Real Sample Study, Cross-Reactivity and Stability Analysis Procedure

The developed sensor’s capability was checked by adding a fixed concentration of meth to different alcoholic and non-alcoholic drinks samples, such as Whisky and Real juice. This solution, together with electrolyte, i.e., 10 mM MB prepared in 0.1 M KCL, was applied to eGAD. Cross-reactivity analysis was also performed by using ketamine (0.01 µg/mL) as an afferent drug on eGAD. Ketamine was deposited onto the apt/AgNPs eGAD, and CV was recorded. Further, the electrochemical assessments were conducted in order to authenticate the results of the meth/apt/AgNPs eGAD tests, which were repeated several times, showing the repeatability of the sensor, and the stability was tested for about 60 days, where the sensor was assayed intermittently on every 10th day.

## 3. Results and Discussion

### 3.1. Characterization of AgNPs

The developed glove-based biosensor depends upon nanomaterials due to their accelerating electron-transporting nature and high surface area. To ensure the biocompatible environment of the sensor, the synthesis and characterization of the synthesized nanoparticles are very crucial steps of the study. Field emission scanning electron microscopy (FE-SEM), Fourier-transform infrared (FTIR) spectroscopy, and UV-Vis spectroscopy, were performed to study the successful synthesis of AgNPs. 

**Figure 3:**
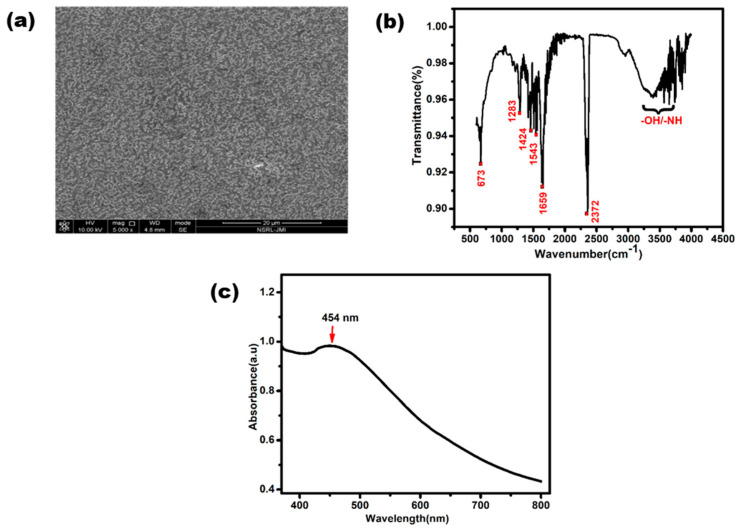
Characterization of silver nanoparticles. (**a**) UV-Vis spectrum of synthesized AgNPs. (**b**) Image of FESEM and (**c**) FTIR spectrum of synthesized AgNPs.

The morphological characterization of the synthesized AgNPs was conducted using FE-SEM. Figure 3a illustrates an image of AgNPs at a scale of 20 µm, revealing their random distribution. The average diameter of these AgNPs had a range of 50–100 nm. Moreover, the observed spherical shape and slight agglomeration of the AgNPs provided further confirmation of their successful formation. FTIR spectroscopy analysis was conducted by scanning the AgNPs in 500–4000 cm^−1^ range with a resolution of 4 cm^−1^. The FTIR spectrum of the synthesized silver nanoparticles is shown in Figure 3b. The peaks present at 3200–3500 cm^−1^ can be assigned to O-H or N-H (amine) stretching. The peaks observed at 2372 cm^−1^ can be attributed to O=C=O (carbonyl bond group) stretching. The peak seen at 1659 cm^−1^ represents the N-H (amine bond). The bands seen at 1424 cm^−1^ and 1283 cm^−1^ represent C-H (alkane bond) and C-O (alcohol/ether) stretching, respectively [35].

Figure 3c shows the UV-Vis absorption spectrum of the synthesized AgNPs. A characteristic peak was clearly observed between 400 to 500 nm, with small hump at exactly about 454 nm due to the surface plasmonic vibration effect. This slight bulge confirms the presence of silver nanoparticles [36]. 

### 3.2. Electrochemical Characterization of Methamphetamine Glove-Based Sensor

**Figure 4:**
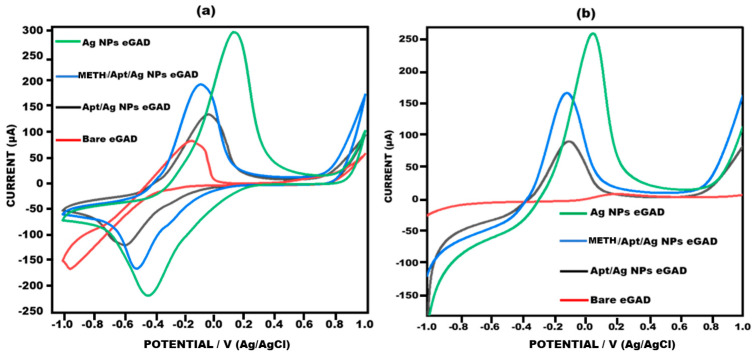
Voltagram profile obtained for bare eGAD, Ag Nps eGAD, Meth/Apt/Ag NPs eGADand Apt/Ag NPs eGAD in 10 mM methylene blue prepared in 0.1 M KCL at 50 mV s^−1^ in the potential range from −1 V to +1 V. (**a**) Cyclic voltammetry. (**b**) Linear sweep voltammetry.

Figure 4 illustrates response of the current observed at different electrode stages, namely bare, AgNPs eGAD, apt/AgNPs eGAD and meth/apt/AgNPs eGAD. This response was validated through potentiostatic techniques, cyclic voltammetry (CV) and linear sweep voltammetry (LSV). The bare electrode demonstrated relatively smaller peak current response during CV and LSV analyses, indicating lower electron transfer kinetics. This can be attributed to the absence of any catalytic material on the electrode surface. However, silver nanoparticle (AgNPs) deposition on the working surface lead to a major two-fold rise in the current response. This enhancement can be attributed to the presence of AgNPs, which act as catalysts and improve the electron transfer kinetics. Subsequently, upon immobilizing the biological recognition element (Aptamer) onto the working surface, a drastic reduction in the current response was witnessed. The reason behind this reduction is the non-conductive nature of aptamer, which hinders the electron transfer process. However, upon target (methamphetamine) introduction, the response of current further rises as compared to that during the aptamer stage. This increase is attributed to the electroactive nature of methamphetamine, which facilitates the electron transfer process and results in an amplified current response.

### 3.3. Analysis Performance of the Developed eGAD

#### 3.3.1. Response Measurement of Developed Glove-Based Sensor

**Figure 5:**
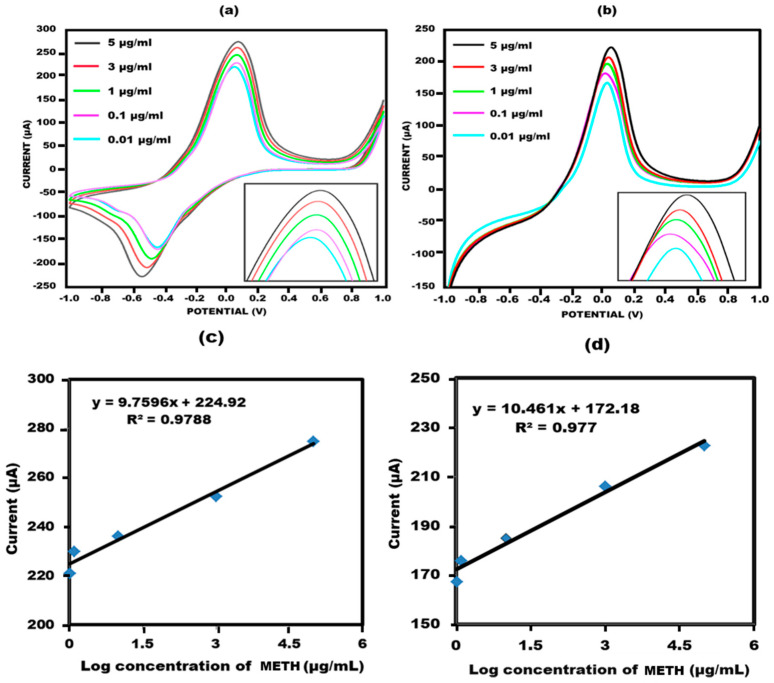
(**a**) Cyclic voltammetry response of the sensor with concentration ranging from 0.01 to 5 µg/mL in 10 mM methylene blue prepared in 0.1 M KCL at 50 mV s^−1^ in the potential range from −1 V to +1 V. (**b**) Linear sweep voltammetry response of the sensor with concentration ranging from 0.01 to 5 µg/mL in 10 mM methylene blue prepared in 0.1 M KCL at 50 mV s^−1^ in the potential ranging from −1 V to +1 V. (**c**) Sensor’s standard deviation for every concentration through cyclic voltammetry. (**d**) Sensor’s standard deviation for every concentration through linear sweep voltammetry.

The developed sensor was used to analyse different concentrations of meth in order to determine its detection range and quantitative performance. To validate the concentration results, two potentiometric techniques, namely cyclic voltammetry (CV) and linear sweep voltammetry (LSV), were performed. Various concentrations of meth were tested, 0.01–5 µg/mL. The results specified that meth exhibits crossing with the aptamer, and different concentrations yielded varying current responses, thereby confirming the quantitative presentation of the sensor. These findings are compatible with earlier reports on similar sensors. As the concentrations of methamphetamine increased, the current response also increased due to the electroactive nature of the compound [37,38,39] (Figure 5a,b).

A noteworthy linear correlation was observed between the peak current value and the logarithm of methamphetamine concentration in cyclic voltammetry, as depicted in Figure 5c. The mathematical representation of this correlation is given by the equation Ia = 9.7596x + 224.92, with an R-squared value of 0.9788. Additionally, the accuracy of the linear sweep voltammetry findings was reaffirmed through linear sweep voltammetry (Figure 5d). A linear connection described by the equation Ia = 10.461x + 172.18 and the associated R-squared value was determined to be 0.977.

#### 3.3.2. Detection Conditions Optimization in Terms of Time and Temperature

**Figure 6:**
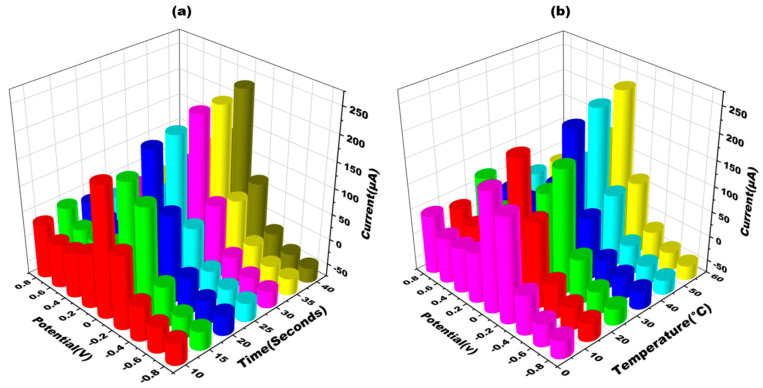
(**a**) Three-dimensional depiction of cyclic voltammograms obtained at METH/apt/AgNPs eGAD for different times (10–40 s) in 10 mM methylene blue prepared in 0.1 M KCL at 50 mV s^−1^ in the potential range from −1 V to +1 V. (**b**) Three-dimensional depiction of cyclic voltammograms obtained at METH/apt/AgNPs eGAD for different temperatures (10–50 °C) in 10 mM methylene blue prepared in 0.1 M KCL at 50 mV s^−1^ in the potential range from −1 V to +1 V.

The sensor’s optimization is one of the crucial step to ensure its appropriate working, as the sensor’s performance can be affected by variations in temperature and time. In order to address this issue, adjustments were made to the sensor’s experimental factors to achieve maximum responsiveness. The developed sensor’s performance was extensively studied across different ranges of temperatures and time durations (Figure 6). The sensor was optimized at various time intervals (in seconds) to determine the maximum response. The current was found to increase from 20 s to 40 s. However, it was determined that the optimum response time was 20 s. At 30 s and 40 s, the current response took longer to manifest, thereby delaying the sensor’s detection capability (Figure 6a). Furthermore, Cyclic voltagrams of the glove-based sensor were recorded at temperatures ranging from 10 °C to 50 °C using a scan rate of 50 mV s^−1^. It was observed that the sensing signal displayed the optimal amplitude at room temperature (approximately 30 °C). Therefore, the sensor was optimized at this temperature to achieve the best performance (Figure 6b). By adjusting the experimental factors of temperature and time, the glove-based biosensor was optimized to ensure its maximum responsiveness and efficient detection capability.

### 3.4. Evaluation Parameters

#### 3.4.1. Limit of Detection/Limit of Quantification and Precision/Recovery Test

The limit of detection and limit of quantification for the developed sensor were found to be 0.1 µg/mL and 0.3 µg/mL, respectively. By using the formula, LOD = 3.3 (Sy/S), and LOQ = 10 (Sy/S), where Sy refers to Standard deviation and S refers to the slope of calibration curve. To evaluate the accuracy of the sensor, a recovery test was conducted. Different concentrations of meth were spiked with known concentration of methamphetamine. For instance, 0.01 µg/mL of meth was added to the samples with other meth concentrations, resulting in a current response that was nearly equivalent to 0.1 µg/mL. The same process was repeated with other concentrations as well. The recovery percentage was then calculated, and the results are presented in Table 1. The recovery percentages obtained were 102% and 103%, respectively, indicating that the proposed biosensor exhibited excellent accuracy and was able to recover the added meth concentrations with very high precision. These results demonstrate the reliability and performance of the suggested biosensor in accurately detecting meth concentrations, making it a promising tool for practical applications.

#### 3.4.2. Cross-Reactivity with Afferent Drug (Ketamine) and Study of Stability, Reproducibility and Repeatability

**Figure 7:**
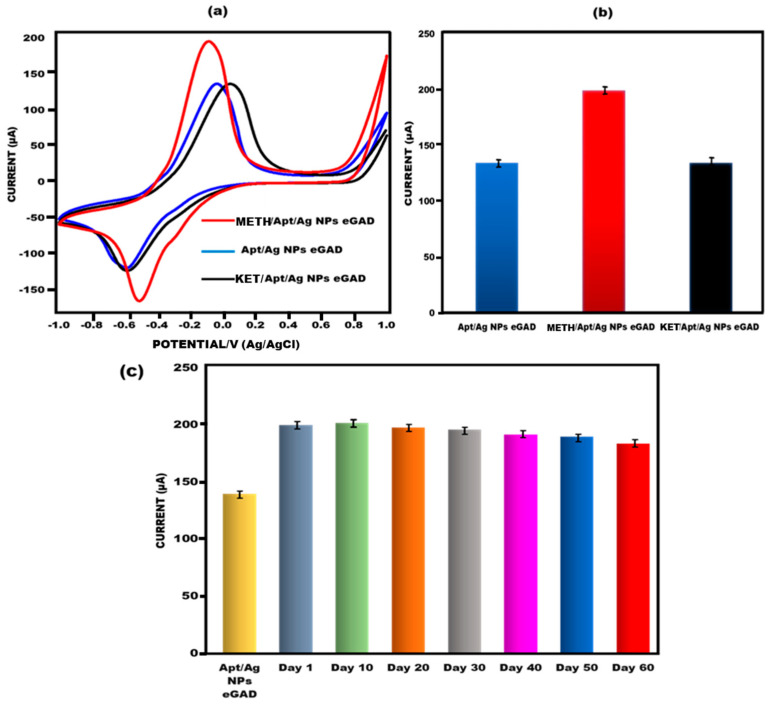
(**a**) Investigation of methamphetamine peak current CVvalue of aptamer/Ag NPs eGAD interaction with the afferent, ketamine. (**b**) Bar graph depicting the interaction of methamphetamine eGAD with afferent ketamine with error bars (n = 5). (**c**) Bar graph depicting how long the built-in electrochemical glove-based analytical device (eGAD) would remain stable.

To assess the cross-reactivity performance of the developed eGAD, a fixed concentration of ketamine (0.01 µg/mL) was used. The current response of the sample was measured using cyclic voltammetry (CV). It can be observed that the peak flow of ketamine is closely equivalent to that of apt/Ag NPs/eGAD, although the current increases when it is compared to that of meth. This indicates the significant cross-reactivity of the sensor towards ketamine (Figure 7a,b). To evaluate the reproducibility of the obtained signals on the developed eGAD, six measurements were performed with a 10-day interval for multiple eGADs that were previously studied. Additionally, the meth eGAD was stored at 4 °C for varying durations (1st day, 10th day, 20th day, 30th day, 40th day, 50th day and 60th day) to detect meth (0.01 µg/mL) and assess its stability using CV. The findings revealed that the sensor remained reliable even until the 60th day, consistently producing results that were almost identical to that of the meth/apt/Ag NPs eGAD, as depicted in Figure 7c. The stability graph also includes error bars, indicating sufficient repetition and reliability for the identification of meth. These results demonstrate that the built-in sensor maintains its reliability and stability over an extended period, making it suitable for the identification and detection of meth with a high degree of accuracy.

### 3.5. Application of Proposed Sensor

**Figure 8:**
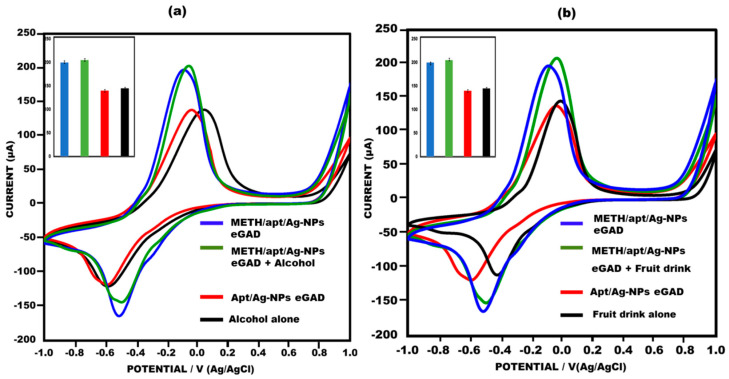
(**a**) Meth in alcohol drink used to construct eGAD in cyclic voltammetry peak current study, with error bars inserted (n = 5); 10 mM methylene blue was prepared in 0.1 M KCL at 50 mV s^−1^ in the potential range from −1 V to +1 V. (**b**) Meth in fruit drink used to construct eGAD in cyclic voltammetry peak current study, with error bars inserted (n = 5); 10 mM methylene blue was prepared in 0.1 M KCL at 50 mV s^−1^ in the potential range from −1 V to +1 V).

To evaluate the practical utility of the developed eGAD, spiked beverages, i.e., An alcoholic drink and a fruit drink were used to examine the effectiveness of the proposed approach in detecting meth. Specifically, 0.01 µg/mL of meth was added to the alcoholic drink and fruit drink, and peak current value testing was performed using cyclic voltammetry on the aptamer/AgNPs NPs eGAD surface.The sensor demonstrated an excellent performance in detecting meth in the spiked alcoholic and fruit drinks, yielding results that were highly comparable to those obtained with meth alone. The data collected met the requirements for accurate identification in a real sample. Notably, the current response observed on the sensor’s surface was found to be nearly identical to that of the meth eGAD alone, indicating the sensor’s ability to successfully detect meth in alcoholic drinks and fruit drinks (Figure 8a,b). These findings highlight the efficiency and practicality of the developed sensor in real-world applications, showcasing its potential for detecting meth in complex samples.

## 4. Conclusions and Future Prospects

Despite significant progress, the field of sensors based on wearable substrates is still in its early stages. Further research and practical applications are crucial for the continued development of such sensors. This study presents, for the first time, an electrochemical glove-based analytical device (eGAD) utilizing silver nanoparticles and aptamers for the sensitive detection of the illicit drug methamphetamine in beverages. Illicit drug abuse, particularly methamphetamine, is a growing concern due to its recreational use and its association with incidents of sexual assault when added to drinks. Electrochemical sensors have garnered considerable interest for their vital role in the early detection of illicit drugs in fluids such as beverages. These sensors offer unique features including specificity, sensitivity, reproducibility, stability, cost-effectiveness, an increased surface-to-volume ratio, improved electron kinetics and rapid response. In this work, the remarkably high charge transfer efficiency of silver nanoparticles was harnessed to develop a detection platform for methamphetamine. The glove-based testing approach provides an affordable point-of-care diagnostics platform. Compared to the current analytical techniques, the proposed sensor requires less time and expense for methamphetamine detection. The synthesized nanoparticles were characterized using FTIR, SEM and UV-vis spectroscopy. Aptamers were chosen as they are highly sensitive and selective tools for rapid diagnostic approaches. The analytical response of the biosensors was measured using cyclic voltammetry (CV) and linear sweep voltammetry (LSV) and validated with a potentiostat. The developed sensor holds promise for monitoring methamphetamine for pharmacokinetic and pharmacodynamic purposes, as well as accurately measuring its presence in spiked samples. It offers a simple, quick and cost-effective solution. Based on the findings of this study, the developed eGAD could be employed as a wearable sensor for drug analysis in the future, contributing to advancements in wearable sensor technology.

## Figures and Tables

**Table 1:** Recovery test depicting the recovery% of the developed meth eGAD.

Primary Concentration (µg/mL)	Spiked Concentration of Meth (µg/mL)	Final Current (µA)	Expected Current (µA)	Recovery Percentage
0.01	0.1	230.6	238.4	103%
0.01	1	240.2	245.8	102%

## Data Availability

Data will be made available on request.
